# Supplementary material for: The red blood cell distribution width is associated with all-cause and cardiovascular mortality among individuals with non-alcoholic fatty liver disease
Source: PLoS One. 2025 Apr 17;20(4):e0321789. doi: 10.1371/journal.pone.0321789 (PMC12005546; doi:10.1371/journal.pone.0321789)
Supplement: Attachment — Submitted filename: Response to Editors.docx [file pone.0321789.s003.docx]

Dear Editor:

We are grateful for the opportunity to revise our manuscript based on your suggestions. We would like to submit our revised manuscript, entitled 'The red blood cell distribution width is associated with all-cause and cardiovascular mortality among individuals with non-alcoholic fatty liver disease' (PONE-D-24-46128R2), for consideration for publication. We have uploaded the data to the Zenodo database (https://doi.org/10.5281/zenodo.14862447). In the revised version, we have revised the Data Availability statement (page 15, line 270-274).

**Data availability statement**

All original data could be publicly available at the NHANES database: <https://wwwn.cdc.gov/nchs/nhanes/Default.aspx>. The data supporting the conclusions of this article have been uploaded to the Zenodo database (https://doi.org/10.5281/zenodo.14862447), without undue reservation.

Should further revisions be required, we are happy to comply. Thank you for your your professional suggestions.

Sincerely,
Ming Hu

Department of Infectious Disease，Beijing Luhe Hospital, Capital Medical University, Beijing, China.

Email: [hmyx2012@sina.com](mailto:hmyx2012@sina.com)
