# Supplementary material for: The red blood cell distribution width is associated with all-cause and cardiovascular mortality among individuals with non-alcoholic fatty liver disease
Source: PLoS One. 2025 Apr 17;20(4):e0321789. doi: 10.1371/journal.pone.0321789 (PMC12005546; doi:10.1371/journal.pone.0321789)
Supplement: Attachment — Submitted filename: Response to Reviewers.docx [file pone.0321789.s002.docx]

Dear Editor and Reviewer:

We appreciate the opportunity to allow us to revise our manuscript and thanks for reviewers’ constructive comments and suggestions. We would like to submit our revised manuscript, entitled “**The red blood cell distribution width is associated with all-cause and cardiovascular mortality among individuals with non-alcoholic fatty liver disease**” (**PONE-D-24-46128**) for consideration for publication. In the revised manuscript, we have carefully addressed all comments and questions raised by reviewer’ point-by-point. We greatly appreciate your time and efforts to improve our manuscript for publication.

**Response to Editor**

1. Please ensure that your manuscript meets PLOS ONE's style requirements, including those for file naming.

Response: Thank you very much for your suggestion. We have thoroughly reviewed the PLOS ONE style templates and have ensured that our manuscript adheres to all specified formatting requirements. We have double-checked the document to ensure consistency with the PLOS ONE guidelines.

2. We noticed you have some minor occurrence of overlapping text with the following previous publication(s), which needs to be addressed:

https://www.frontiersin.org/journals/nutrition/articles/10.3389/fnut.2024.1467824/full

In your revision ensure you cite all your sources (including your own works), and quote or rephrase any duplicated text outside the methods section. Further consideration is dependent on these concerns being addressed.

Response: Thank you for your valuable feedback. We apologize for the overlap with our published article (PMID: 39421611) and have revised the Methods section to rephrase the content (page 8, line 138-151).

**Evaluation of mortality from all causes and follow-up**

Mortality status was determined by integrating information obtained from the National Death Index, which may be reached at <https://www.cdc.gov/nchs/data-linkage/mortality-public.htm>, with the NHANES data. Participants were classified as either alive or deceased based on NDI data. The follow-up period was calculated by subtracting the date of the NHANES examination from the date of death (December 31, 2019). Cardiovascular and all-cause mortality were retrieved and analyzed using the 10th Revision of the International Classification of Diseases (ICD-10). Cardiovascular mortality was defined as death due to cardiac conditions, which were categorized under the codes I00–I09, I11, I13, and I20–I51. The median follow-up period was 124 months (interquartile range: 100–147).

3. PLOS requires an ORCID iD for the corresponding author in Editorial Manager on papers submitted after December 6th, 2016. Please ensure that you have an ORCID iD and that it is validated in Editorial Manager. To do this, go to ‘Update my Information’ (in the upper left-hand corner of the main menu), and click on the Fetch/Validate link next to the ORCID field. This will take you to the ORCID site and allow you to create a new iD or authenticate a pre-existing iD in Editorial Manager.

Response: Thank you for the detailed instructions provided. We have confirmed that the corresponding author (Ming Hu) now has an ORCID ID (0009-0002-4899-6751), which has been validated in Editorial Manager.

4. Your ethics statement should only appear in the Methods section of your manuscript. If your ethics statement is written in any section besides the Methods, please move it to the Methods section and delete it from any other section. Please ensure that your ethics statement is included in your manuscript, as the ethics statement entered into the online submission form will not be published alongside your manuscript.

Response: Thank you for your guidance regarding the placement of the ethics statement in our manuscript. We have reviewed our manuscript and ensured that the ethics statement now appears only in the Methods section. Any mention of the ethics statement in other sections has been removed.

**Response to reviewer**

1.The entire manuscript requires editing by a native English speaker for clarity and fluency.
Response: Thank you for your valuable suggestions. We fully agree that a native English speaker's input can significantly enhance the readability and coherence of our work. In response to your suggestion, we have engaged a professional native English editor with expertise in academic writing. This editor has carefully reviewed and revised the entire manuscript to ensure that the language is clear, concise, and fluent.

2. The logical structure of the Introduction is not sufficiently clear, which may be due to language issues. For instance, the second and third paragraphs redundantly introduce RDW. A review of published literature indicates that there are numerous studies focused on the relationship between RDW and NAFLD, contradicting the authors’ claim that “There were few researches explored the relationship between RDW on NAFLD.”
Response: We greatly appreciate the reviewer’s insightful comment. We acknowledge that the redundancy between the second and third paragraphs could lead to confusion, and we have revised this section to improve clarity and eliminate repetition (paragraph 2).

Red blood cell distribution width (RDW), a routinely measured parameter in a complete blood count (CBC), assesses the variation in the size of red blood cells (RBCs). RDW is calculated by determining the standard deviation of RBC volume relative to mean corpuscular volume (MCV). The normal range for RDW is typically 11–15%[7]. While low RDW values are generally not clinically significant, elevated RDW levels can be associated with increased levels of inflammatory markers such as erythrocyte sedimentation rate (ESR), C-reactive protein (CRP), and interleukins (IL)[7]. Numerous studies have demonstrated that RDW can serve as a prognostic marker for various diseases, including acute myocardial infarction[8], covid-19[9], heart failure[10], sepsis[11], and hepatocellular carcinoma[12]. Although several studies have explored the association between RDW and various conditions, fewer have focused on its relationship with NAFLD, Yang *et al*. [13]reported that patients with NAFLD tended to exhibit elevated RDW levels in a Chinese hospital cohort. However, in the United State, the relationship between RDW and mortality in patients with NAFLD remains unclear.

3.In the Study Design and Participants section, the authors define excessive alcohol consumption as more than 4 cups per day for men and more than 3 cups for women. What is the basis for these definitions? What type of alcoholic beverages are included? These standards are crucial for accurately including patients with non-alcoholic cirrhosis.
response: Thank you for your thoughtful comment. We understand the importance of clearly defining these parameters to ensure accurate inclusion of patients with non-alcoholic cirrhosis. And we revised the manuscript with clear definition of excessive alcohol intake.

(1).The definition of excessive alcohol consumption in our study is based on established references in the literature(16): excessive alcohol consumption as more than 4 drinks per day for men and more than 3 drinks for women. excessive alcohol intake[16] (≥4 drinks per day for men and ≥3 drinks per day for women; one drink is defined as a 12 ounce beer, a 5 ounce glass of wine, or 1.5 ounces of liquor, including whisky, gin, beer, wine, wine coolers, and any other alcoholic beverage (page 6, line 86-90)

(2). What type of alcoholic beverages are included?

Alcoholic beverages included are whiskey, gin, beer, wine, wine coolers, and any other alcoholic beverage (page 6, line 88-89).

4.Serum levels of CRP, TCHO, ALT, GLU, TG, SCr, and HDL-C should be included in the analysis model. Additionally, since the authors propose that RDW is an important inflammatory marker, it would be beneficial to provide correlation data between RDW and CRP.

(1) Response: We appreciate your thoughtful suggestions. Regarding the inclusion of CRP in the analysis model, we would like to clarify that CRP data is available only in the 2005-2010 NHANES cycles, and is missing in the 2011-2016 cycles. As a result, including CRP would lead to incomplete data for the cohort, and we were unable to incorporate it into our analysis model. While we acknowledge that CRP is an important inflammatory marker, the absence of data in the later cycles made it unfeasible to include it. Additionally, we chose not to include triglycerides (Tg) in the final model due to concerns about multicollinearity, as TG is closely associated with the definition of NAFLD. After careful consideration during model development, we excluded Tg to avoid potential issues arising from collinearity between predictors.

However, we have included other relevant serum markers, such as TCHO, ALT, GLU, SCr, and HDL-C, as part of a sensitivity analysis to provide a comprehensive understanding of the factors influencing RDW and its association with clinical outcomes. The sensitivity analysis results demonstrated that RDW was associated with both all-cause mortality and cardiovascular mortality (P<0.05) (Table 3).

Table 3 The relationships between RDW and mortality in NALFD, additionally adjusted for TCHO, ALT, GLU, SCr, and HDL-C

| Characteristic | Crude model | | Adjusted model | |
| --- | --- | --- | --- | --- |
|  | HR(95% CI) | P value | HR(95% CI) | P value |
| All-cause mortality | | | | |
| RDW | 1.30 (1.24-1.36) | <0.001 | 1.21(1.13-1.30) | <0.001 |
| RDW quartile | |  |  |  |
| Q1(≤12.2) | Reference |  | Reference |  |
| Q2(12.3-12.7) | 1.73 (1.30-2.31) | <0.001 | 1.38 (1.03-1.85) | 0.032 |
| Q3(12.8-13.4) | 2.92 (2.22-3.85) | <0.001 | 1.58 (1.22-2.04) | <0.001 |
| Q4(≥13.5) | 4.87 (3.68-6.35) | <0.001 | 2.20(1.64-2.95) | <0.001 |
| P for trend | | <0.001 |  | <0.001 |
| Cardiovascular mortality | | | | |
| RDW | 1.33 (1.27-1.39) | <0.001 | 1.21 (1.12-1.31) | <0.001 |
| Q1 (≤12.2) | Reference |  | Reference |  |
| Q2 (12.3-12.7) | 2.60 (1.60-4.24) | <0.001 | 2.06 (1.24-3.43) | 0.005 |
| Q3 (12.8-13.4) | 4.32 (2.63-7.08) | <0.001 | 2.19 (1.31-3.66) | 0.003 |
| Q4 (≥13.5) | 8.36 (5.32-13.13) | <0.001 | 3.44(2.06-5.75) | <0.001 |
| P for trend | | <0.001 |  | <0.001 |

Adjusted model: adjusted for sex, age, race, marriage, education, smoke, BMI, CVD, diabetes, hypertension, TCHO, ALT, GLU, SCr, and HDL-C.

RDW: red blood cell distribution width; HR: hazard ratio.

(2) Additionally, since the authors propose that RDW is an important inflammatory marker, it would be beneficial to provide correlation data between RDW and CRP.

Response: Thank you for your valuable suggestion. We acknowledge that CRP is a well-established inflammatory marker, and examining its correlation with RDW could strengthen our argument regarding the inflammatory role of RDW in NAFLD.

To address this, we conducted a linear regression analysis assessing the association between RDW and CRP with data from 2005-2010 years. The results demonstrated RDW was positively associated with CRP (**β 0.16, 95%CI: 0.12-0.21, P<0.001**) (Table S1).

Table S1 The association between RDW and CRP

| Variable | N | Crude model | | Adjusted model | |
| --- | --- | --- | --- | --- | --- |
|  |  | Coefficient (95%CI) | P value | Coefficient (95%CI) | P value |
| RDW | 5740 | 0.14 (0.12~0.15) | <0.001 | 0.16 (0.12~0.21) | <0.001 |

Adjusted for age, sex, race, marital status, education, BMI, smoking status, alcohol use, CVD, diabetes, and hypertension.

5.The vertical axes in Figures 2A and 2B should be labeled as All-Cause Mortality and Cardiovascular Mortality, respectively.

Response: Thank you for pointing this out. We have revised Figures 2A and 2B to ensure that the vertical axes are correctly labeled as "A All-Cause Mortality" and "B Cardiovascular Mortality," respectively.

6.The Discussion section is overly simplistic, particularly in its insufficient exploration of the relationship between RDW and cardiovascular mortality.

Response: Thank you for your insightful comment. We acknowledge that the relationship between RDW and cardiovascular mortality was not fully explored in the original manuscript. Based on your suggestion, we have revised the Discussion section to provide a more in-depth analysis of this relationship (page12-13, line230-239).

Previous research has demonstrated that elevated RDW is associated with cardiovascular mortality[28]. [Katamreddy](https://pubmed.ncbi.nlm.nih.gov/?term=Katamreddy+A&cauthor_id=35229542) *et al.*[29] reported that an elevated RDW was associated with cardiovascular mortality in the intermediate ASCVD group in a study of  8884 subjects from NHANES III. Liao *et al.*[30] found that RDW was an independent predictor and showed a linear association with 1-year cardiovascular mortality in patients undergoing percutaneous coronary intervention (PCI). Lin *et al.*[31] identified RDW as an independent predictor of cardiovascular mortality in 181 STEMI patients (OR = 1.288, 95% CI, 1.126–1.472; P = 0.0005). This study’s findings indicate that RDW is associated with cardiovascular mortality in the NAFLD population, aligning with the results reported above.

**Reference**

16. Zhao E, Cheng Y, Yu C, Li H, Fan X. The systemic immune-inflammation index was non-linear associated with all-cause mortality in individuals with nonalcoholic fatty liver disease. Ann Med. 2023 Dec;55(1):2197652.
